# Visual outcomes 24 months after LaserACE

**DOI:** 10.1186/s40662-017-0081-y

**Published:** 2017-06-16

**Authors:** AnnMarie Hipsley, David Hui-Kang Ma, Chi-Chin Sun, Mitchell A. Jackson, Daniel Goldberg, Brad Hall

**Affiliations:** 1 Ace Vision Group Inc, 39655 Eureka Drive, Newark, CA 94560 USA; 2 Department of Ophthalmology, Chang Gung Memorial Hospital, Kweishan, Taoyuan Taiwan; 3 Department of Chinese Medicine, Chang Gung University, Kweishan, Taoyuan Taiwan; 4 Center for Tissue Engineering, Chang Gung Memorial Hospital, Kweishan, Taoyuan Taiwan; 5 Department of Ophthalmology, Chang Gung Memorial Hospital, Keelung, Taiwan; 6 Jackson Eye, Lake Villa, IL USA; 7 Drexel College of Medicine, Philadelphia, PA USA; 8 Sengi Data, Cambridge, ON Canada

**Keywords:** Presbyopia, Accommodation, Clinical trial, Asian eyes, Visual acuity

## Abstract

**Background:**

To evaluate the effects on near and intermediate visual performance after bilateral Laser Anterior Ciliary Excision (LaserACE) procedure.

**Methods:**

LaserACE surgery was performed using the VisioLite 2.94 μm erbium: yttrium–aluminum–garnet (Er:YAG) ophthalmic laser system in 4 oblique quadrants on the sclera over the ciliary muscle in 3 critical zones of physiological importance (over the ciliary muscles and posterior zonules) with the aim to improve natural dynamic accommodative forces. LaserACE was performed on 26 patients (52 eyes). Outcomes were analyzed using visual acuity testing, Randot stereopsis, and the CatQuest 9SF patient survey.

**Results:**

Binocular uncorrected near visual acuity (UNVA) improved from +0.20 ± 0.16 logMAR preoperatively, to +0.12 ± 0.14 logMAR at 24 months postoperatively (*p* = 0.0014). There was no statistically significant loss in distance corrected near visual acuity (DCNVA). Binocular DCNVA improved from +0.21 ± 0.17 logMAR preoperatively, to +0.11 ± 0.12 logMAR at 24 months postoperatively (*p* = 0.00026). Stereoacuity improved from 74.8 ± 30.3 s of arc preoperatively, to 58.8 ± 22.9 s of arc at 24 months postoperatively (*p* = 0.012). There were no complications such as persistent hypotony, cystoid macular edema, or loss of best-corrected visual acuity (BCVA). Patients surveyed indicated reduced difficulty in areas of near vision, and were overall satisfied with the procedure.

**Conclusions:**

Preliminary results of the LaserACE procedure show promising results for restoring visual performance for near and intermediate visual tasks without compromising distance vision and without touching the visual axis. The visual function and visual acuity improvements had clinical significance. Patient satisfaction was high postoperatively and sustained over 24 months.

**Trial registration:**

NCT01491360 (https://clinicaltrials.gov/ct2/show/NCT01491360). Registered 22 November 2011.

## Background

Presbyopia has traditionally been defined as the gradual loss of accommodation resulting from loss of elasticity of the lens capsule and lens substance only [1]. Hemholtz’ theory of accommodation described how the ciliary muscles contract during accommodative effort, releasing tension on the anterior zonules, and allowing the elastic lens capsule to reshape and change the dioptric power of the lens [2]. An inelastic lens would therefore reduce accommodation, resulting in presbyopia [2]. Under this model, treatment options could involve spectacles, contact lenses, and surgical correction. Surgical correction could be done with either corneal refractive surgery or intraocular lens replacement [3]. Corneal refractive procedures include excimer ablation to create monovision or multifocality, conductive keratoplasty using radiofrequency waves, and inlays [4]. Intraocular lens replacement uses monofocal lenses for monovision, multifocal implants, accommodative implants, and most recently, extended depth of focus (EDOF) intraocular implants [4]. Of these modalities, only intraocular accommodative lenses attempt to restore accommodation to the presbyopic eye [5]. Also, corneal presbyopic procedures carry risks of scarring, night vision problems and vision loss, and lenticular procedures carry risks of endophthalmitis and night vision problems [6].

Recent research has demonstrated the important role of the extralenticular structures (including the ciliary body, zonules, anterior vitreous membrane, and elastic foundation in the choroid), which have added new direction to the surgical treatments of presbyopia [7–10]. Using ultrasound biomicroscopy and endoscopy [7, 8], optical coherence tomography [10], and magnetic resonance imaging [9], changes in the vitreous membrane, peripheral choroid, ciliary muscle, and zonules, as well as the effects of aging, have been documented. It has also been shown that the sclera bows inward with increasing age [8]. The loss of accommodation may be better described by accommodative lens thickening and resting muscle apex thickness together, rather than by lens thickening solely [11]. Stiffening of the zonular apparatus may also contribute to loss of accommodation [12]. Ocular rigidity has also been correlated with aging and the loss of accommodation, which have clinical significance [13]. Finally, the role of proprioceptors in the vitreous zonular system has been identified and supports the premise that biomechanical dysfunction impacts the neuromuscular system of accommodation and the declining efficiency of accommodative forces [14]. This further establishes a need for interventions, both surgical and therapeutic, to restore functional biomechanics in the accommodation apparatus.

**Fig. 1:**
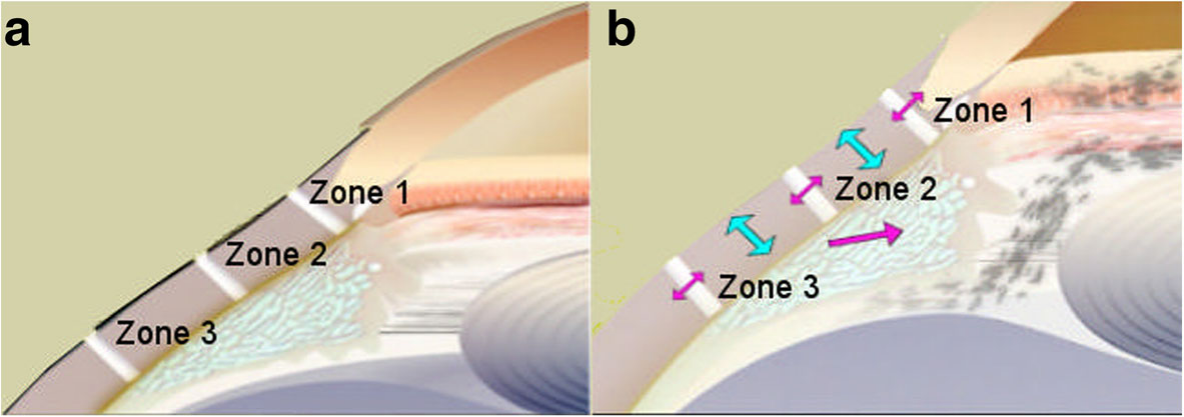
LaserACE surgical procedure. **a** the three critical zones of significance as measured from the anatomical limbus; **b** restored mechanical efficiency and improved biomechanical mobility (procedure objectives)

The human sclera loses elasticity with age [15]. Ocular rigidity has been correlated with loss of accommodation and has been found to have clinical significance for age-related dysfunction of the eye [13]. In addition, the normal inward and upward bowing of the sclera upon accommodative force decreases with age [7]. Laser anterior ciliary excision (LaserACE) is designed to alter biomechanical properties and restore compliance to rigid ocular tissue by creating 9 micropores (600 μm in diameter) in a matrix, in the four oblique quadrants of the eye, and over three critical zones of anatomical and physiological significance [7, 8, 12, 16–19]. Hipsley proposed these 3 critical zones of anatomical and physiological significance to restore accommodative movements and to promote biomechanical efficiency that were later validated by in vivo studies [7, 8, 12, 16–19]. These studies have shown that during accommodation, the sclera moves inward and upward (anteriorly and centripetally) [7, 8]. Also, the ciliary apex moves forward toward the lens, which decreases the circumlental space (Zone 1) [7, 8]. This facilitates the force of the ciliary muscle apex at the scleral spur and longitudinal muscle. Additionally, by measuring changes in distance between the scleral spur and the vitreous zonule insertion zone, the vitreous zonule insertion zone has been shown to move forward during accommodation [12, 19]. The choroid also moves forward during accommodation (Zone 2) [8]. Furthermore, the posterior insertion zone of the vitreous zonules move forward in a sagittal plane along the curvilinear boundary of the globe (anteriorly toward the scleral spur) during accommodation (Zone 3) [19]. This forward movement correlates with accommodative amplitude, and greater forward movement leads to higher accommodative amplitude. The forward movement of the posterior vitreous zonule insertion zone declines with age, as does the space between the vitreous membrane and the ciliary body [12]. Thus, in alignment with recent literature findings regarding locations of accommodative structures of critical importance, the 3 treatment zones are as follows and range from 0.5 mm up to 6.0 mm from the anatomical limbus (AL): 1) the scleral spur at the origin of the ciliary muscle (0.5 - 1.1 mm from AL); 2) the mid ciliary muscle body (1.1 – 4.9 mm from AL); and 3) insertion of the longitudinal muscle fibers of the ciliary, just anterior to the ora serrata at the insertion of the posterior vitreous zonules (4.9 – 5.5 mm from AL) [8, 12, 16, 17, 19]. The matrix array of micropores creates regions in the rigid sclera, which contain areas of both positive stiffness (remaining interstitial tissue) and negative stiffness (removed tissue or micropores). This type of arrangement of the laser-created micropores renders the viscoelastic modulus of the treated scleral regions more compliant when subjected to force or stress, such as contraction of the ciliary muscles [20]. Subsequently, the treated regions of the micropore mesh are highly capable of plasticity and aim to produce a dampening effect when the ciliary muscles exert force. With a more compliant sclera, the distance from the scleral spur to the posterior insertion zone becomes shorter, and the accommodative ciliary muscle contraction results in enhanced anterior and centripetal movement of the ciliary apex, which allows increased movement of the anterior zonule and greater lenticular accommodation [21]. In effect, the reduced scleral rigidity from treatment compensates for the loss of elasticity in the choroid where the posterior zonules insert. Therefore, the proposed mechanism of action of LaserACE is to increase plasticity and compliance of scleral tissue by creating these regions of micropore mesh over the ciliary complex, thereby improving biomechanical function and efficiency of the accommodation apparatus (Fig. 1).

In a previous multicenter international study (Mexico, Canada, Europe, South America) 134 eyes of 67 patients received the LaserACE procedure [22]. These studies were performed serially in each location, iterating the procedure 7 times. The 9-spot matrix was found to be the safest technique and achieved the desired effect without affecting the corneal refractive status. This 9-spot pattern is evaluated in this study. We believe this to be the first long term report of use of LaserACE to restore near and intermediate visual performance. Twenty-four-month data obtained from a clinical trial is reported.

## Methods

A prospective, non-comparative study was approved at the Chang Gung Memorial Hospital, Linkou, Taiwan. This was an Institutional Review Board (IRB) monitored and registered international clinical pilot study approved by IRB.GOV, and followed the tenets of the Declaration of Helsinki and local Taiwan healthcare laws. After a full explanation of the purpose of the study and the LaserACE procedure, signed informed consent was obtained from all patients. All study participants agreed to return for the postoperative examinations. Two surgeons (DHKM and CCS) performed all procedures. Inclusion criteria included patient age ≥ 40 years, and healthy eyes with a demonstrated loss of accommodative function. Participants had less than 1.00 D of astigmatism measured in their manifest refraction in each eye, and corrected distance visual acuity (CDVA) equal to or better than 20/40 in each eye. Less than 0.50 D difference existed between manifest and cycloplegic refraction. Laser vision correction patients were included (*N* = 4). Patients were excluded from this study if they were pregnant or breast feeding, had previous ocular surgeries other than laser vision correction, or had a history of scleral ectasia, scleritis, or episcleritis. Patients were accepted if they had an intraocular pressure (IOP) between 11 and 30 mmHg and were not prescribed pressure lowering medications. Fifty-two eyes of 26 patients underwent the Laser Anterior Ciliary Excision (LaserACE) procedure.

### Preoperative and postoperative assessments

The patients had a thorough eye examination including objective and manifest visual acuity, IOP (pneumatic tonometer), pupil size (neuroptics pupilometer), keratometric measurements, slit lamp evaluation, stereoacuity (Randot stereoscopic test), wavefront aberrometry (Tracey Technologies), and fundoscopy. Regular topographic patterns of the front and back cornea were confirmed with a Pentacam-HR Scheimpflug camera (Oculus, Inc.). Central corneal thickness was measured with an optical low-coherence reflectometry pachymeter and the Pentacam-HR tomographer. Scleral thickness was measured for safety with dynamic, high-definition ultrasound biomicroscopy (Sonomed Escalon) and only eyes with a calculated preoperative scleral thickness of 400 μm or more were included.

### Visual acuity

Illuminated early treatment diabetic retinopathy study (ETDRS) charts were used to assess visual acuity at distance (4 m; 100% contrast ETDRS chart), intermediate (60 cm; ETDRS visual acuity chart 2), and near (40 cm; ETDRS visual acuity chart 1). Patients read down the chart slowly, row by row, beginning with the first letter on the top row. When the patients had difficulty reading a letter they were encouraged to guess. The test ended when it was evident that no further meaningful letter could be identified, despite urging the subject to guess. Correctly read letters were recorded on a score sheet with an identical layout to that of the chart. The log minimum angle of resolution (logMAR) score was calculated by adding the logMAR of the best-read line to 0.1 logMAR, and subtracting 0.02 logMAR units for each letter read. Photopic lighting conditions were 85-90 cd/m^2^.

### Device and surgical methods

**Fig. 2:**
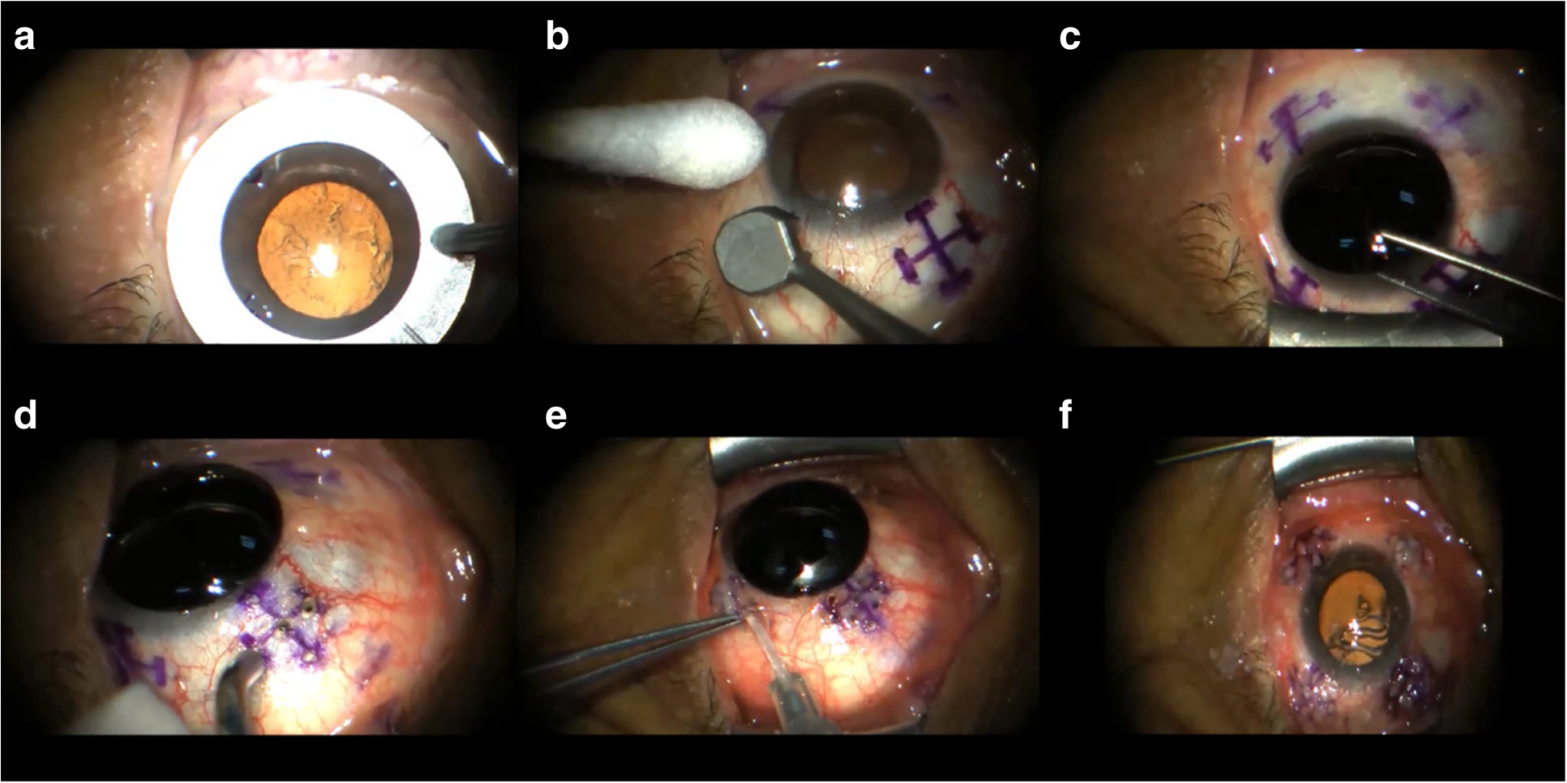
LaserACE surgical technique. Photo **a** Quadrant marker; **b** Matrix marker; **c** Corneal Shield; **d** LaserACE micropore ablation; **e** Subconjunctival Collagen; **f** Completed 4 quadrants

An overview of the LaserACE surgical technique is shown in Fig. 2. Two experienced LaserACE surgeons performed all procedures bilaterally on the same day. Prior to the procedure, topical tobramycin/dexamethasone, and tetracaine, or equivalents of any of these three eye drops, as well as diazepam or alprazolam orally, were administered. Patients also received 1 drop of brimonidine 0.15% every 10 min for 3 doses over 30 min prior to surgery to reduce bleeding. Tetracaine and a third or fourth generation fluroquinolone were applied to the cornea prior to the procedure. An opaque corneal shield was placed on the cornea, and remained in place until the completion of the procedure.

An erbium: yttrium–aluminum–garnet (Er:YAG) laser (VisioLite) was utilized to create micropores in the sclera. The laser frequency was 10-30 Hz and laser fluence was 30-50 mJ/cm^2^. The spot size was 600 μm, delivered through a fiber hand piece and near-contact 80° curved tip. Excisions were placed in a matrix pattern from 0.5 mm from up to 6.0 mm from AL over the 3 critical anatomical and physiological zones of significance. Excision depth was 85-90% the depth of the sclera, to the point that the blue hue of the choroid just became visible. Each ablation began with a faster frequency of 30 Hz, and slowed down to 10 Hz when approaching the deeper layer so as not to penetrate the choroid.

A Collagen Matrix powder (Collawound, Collamatrix) was mixed with a ratio of 1:4 (*v*/v) sterile saline solution in a 10 mL syringe and applied directly over the scleral ablation matrices with a cannula. An 18 mm scleral contact lens was routinely used postoperatively to cover the ablation zones and hold the collagen in place. Topical antibiotics and steroids were used in both eyes, 4 times a day for 7 days, followed by a steroid taper.

Patients were evaluated postoperatively on days 1, 3, 7, and after 1, 3, 6, 12, 18, and 24 months.

### Patient-reported visual function

The CatQuest 9SF Survey was used to investigate patient satisfaction and patient-reported visual function preoperatively and postoperatively at 6, 18, and 24 months [23].

### Statistical analysis

Data were analyzed using repeated-measures analysis of variance (ANOVA). Tukey honestly significant difference post hoc comparisons were performed, where applicable. A *p* < 0.05 was taken to be significant. The measurements obtained at 1, 3, 6, 12, and 24 months were included in the statistical tests.

## Results

### Demographics and surgical information

Twenty-six subjects were enrolled, ranging in age from 45 to 64 years, and a mean age of 49.7 ± 4.37 years. Twenty-one patients completed 24 months of postoperative care. Five patients withdrew, due to occupational travel conflicts. Four patients were S/P laser vision corrected while the remainders were naturally emmetropic (Table 1). Visual outcomes in this study appeared to be very sensitive to corneal refractive status. To understand the specific effects on visual acuity, we chose to narrow the definition of ‘emmetropic’ by 0.25 D steps. Therefore, any patient that was close to 0 or between −0.25 D to +0.25 D we defined as true Emmetropes and anything beyond −0.25 D to −0.5 D we defined as emmetropic myopes. Likewise, any patient between +0.25 D to +0.5 D we defined as emmetropic hyperopes.

**Table 1:** Preoperative patient demographics

	Emmetrope	Emmetropic Myope	Emmetropic Hyperope	LVC Myope
N	6	6	10	4
Gender	Male = 66.7% Female = 33.3%	Male = 66.7% Female = 33.3%	Male = 30.0%, Female = 70.0%	Male = 25.0% Female = 75.0%
Spherical Equivalent Refraction (mean ± SD)	0	−0.30 ± 0.11 D	0.50 ± 0.25 D	0.25 ± 0.35 D
Cylinder (mean ± SD)	−0.17 ± 0.14 D	−0.15 ± 0.22 D	0.00 ± 0.22 D	−0.19 ± 0.38 D
Age (Median)	49	50	49	45
CDVA ≥20/25	100%	100%	100%	100%
CDVA ≥20/20	100%	100%	100%	100%
CDVA ≥20/16	100%	100%	80%	100%

*LVC* laser vision corrected, *CDVA* corrected distance visual acuity

### Uncorrected visual acuity

**Fig. 3:**
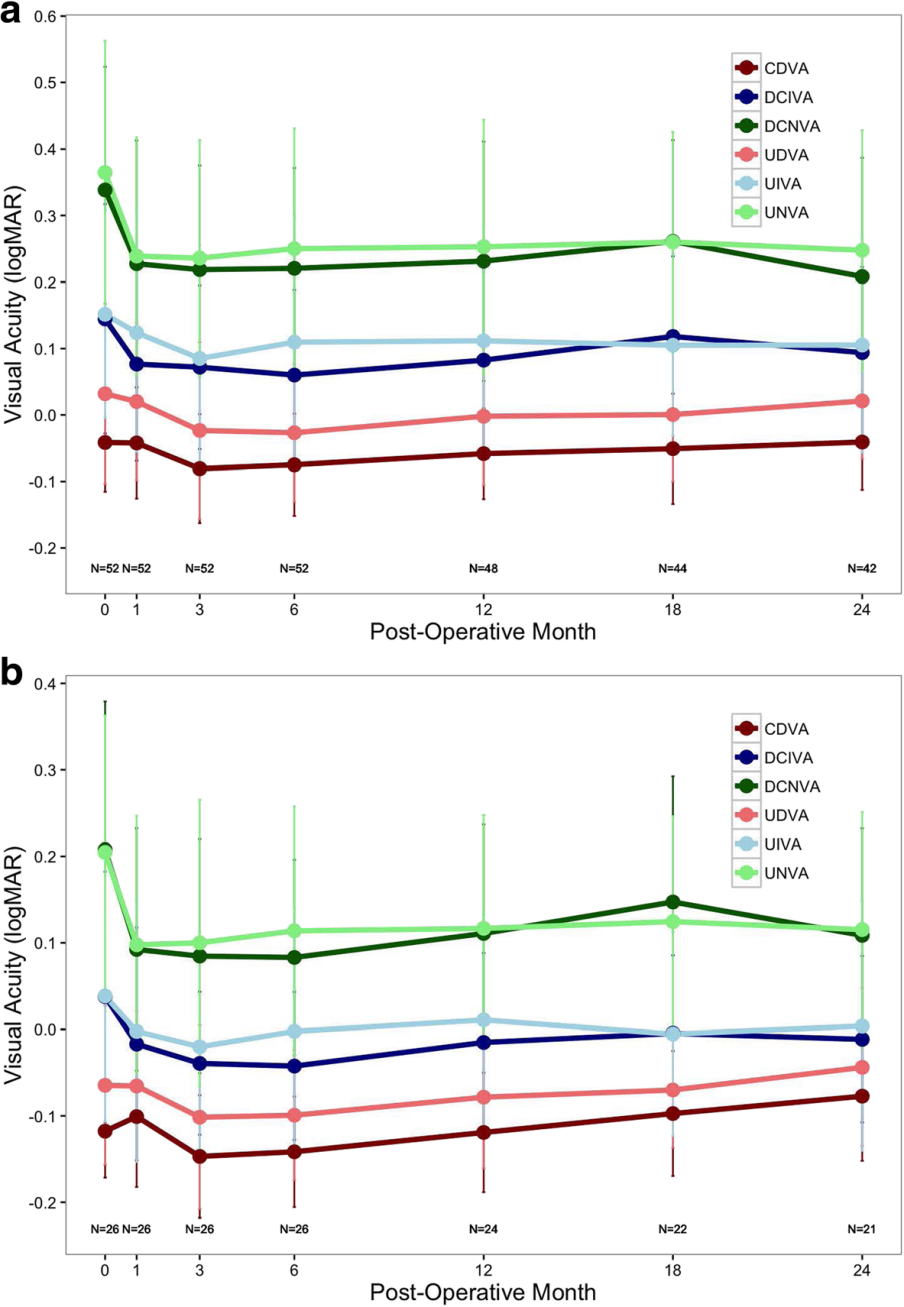
Uncorrected (*lightly colored*) and distance-corrected (*darkly colored*) visual acuity at distance (4 m) intermediate (60 cm), and near (40 cm) for **a**) monocular and **b**) binocular patient eyes. Error bars represent mean ± SD

Preoperative and postoperative monocular uncorrected visual acuity (UVA) logMAR are shown in Fig. 3. The largest improvements in visual acuity overall were for monocular uncorrected near visual acuity (UNVA) measured at 40 cm. Mean monocular UNVA for all patients was significantly improved at all follow-up visits and was 0.25 ± 0.18 logMAR (~20/35 snellen) at 24 months postoperatively compared to preoperative monocular UNVA of 0.36 ± 0.20 logMAR (~20/45 snellen) (*p* = 0.000050). Binocular UNVA improved from +0.20 ± 0.16 logMAR (~20/32 snellen) preoperatively, to +0.12 ± 0.14 logMAR (~20/25 snellen) at 24 months postoperatively (*p* = 0.0014).

Monocular uncorrected intermediate visual acuity (UIVA) measured at 60 cm increased postoperatively for all time points compared to preoperative UIVA, and was statistically significant at 3 months postoperatively (*p* = 0.0040). At 24 months postoperatively, there was no statistically significant loss or change from preoperative refraction. Similar to UIVA, monocular uncorrected distance visual acuity (UDVA) measured at 4 m increased at all time points and was statistically significant at 3 and 6 months postoperatively (*p* = 0.0080 and *p* = 0036). Binocular UIVA increased for all points compared to preoperative UIVA and was statistically significant at 3 months postoperatively (*p* = 0.0047). At 24 months postoperatively, binocular UDVA showed no statistically significant loss or change from preoperative refraction.

### Distance corrected visual acuity

Preoperative and postoperative monocular distance corrected visual acuity (DCVA) in logMAR are shown in Fig. 3. Similar to UVA, the largest improvements in visual acuity were for distance corrected near visual acuity (DCNVA) measured at 40 cm. Mean monocular DCNVA for all patients was significantly improved (*p* = 0.000000019) at all follow-up visits and was 0.21 ± 0.18 logMAR (~20/32 snellen) at 24 months postoperatively compared to preoperative monocular DCNVA of 0.34 ± 0.18 logMAR (~20/45 snellen). Binocular DCNVA improved from +0.21 ± 0.17 logMAR (~20/32 snellen) preoperatively, to +0.11 ± 0.12 logMAR (~20/25 snellen) at 24 months (*p* = 0.00026).

Monocular distance corrected intermediate visual acuity (DCIVA) measured at 60 cm increased postoperatively for all time points compared to preoperative DCIVA, and was statistically significant at 1, 3, 6, and 12 months postoperatively (*p* = 0.0019, *p* = 0.00065, *p* = 0.000031, and *p* = 0.0087). At 24 months postoperatively there was no statistically significant loss or change from preoperative refraction. Similar to DCIVA, monocular corrected distance visual acuity CDVA measured at 4 m increased at all time points and was statistically significant at 3 months postoperatively (*p* = 0.015). Binocular DCIVA increased for all points compared to preoperative DCIVA and was statistically significant at 1, 3, 6, and 12 months postoperatively (*p* < 0.0087). At 24 months postoperatively, binocular CDVA showed no statistically significant loss or change from preoperative refraction.

### Stability, intraocular pressure, and stereopsis

**Fig. 4:**
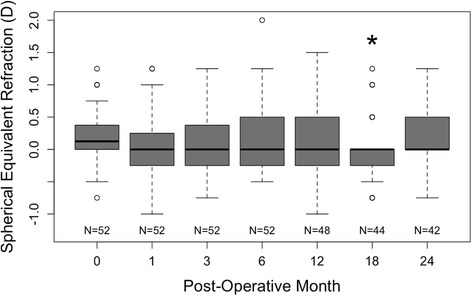
Box-and-whiskers plot of the stability of the spherical equivalent refraction of patient eyes. The upper and lower extremities of the box represent the 75 and 25th percentiles, the bar within the box represents the median, the whiskers represent the full extent of the data ranges, and the points represent outliers. The star denotes statistical significance compared to preoperative values

The spherical equivalent refraction, shown in Fig. 4, was stable over 24 months operatively. At 18 months postoperatively, the spherical equivalent refraction was significantly improved at 0.00 ± 0.46 D compared to preoperative refraction 0.16 ± 0.42 D (*p* = 0.0015).

**Fig. 5:**
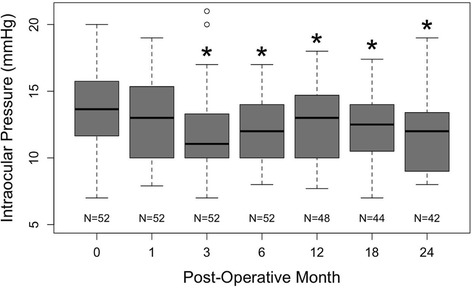
Box-and-whiskers plot of the postoperative changes in intraocular pressure (IOP) of patient eyes. The upper and lower extremities of the box represent the 75 and 25th percentiles, the bar within the box represents the median, the whiskers represent the full extent of the data ranges, and the points represent outliers. The stars denote statistical significance compared to preoperative values

Intraocular pressure (IOP) as measured by pneumatic tonometry is shown in Fig. 5. Patient IOP averaged of 13.56 ± 3.23 mmHg preoperatively. Patient IOP was significantly lower than preoperative IOP at all time points apart from 1 month postoperatively (*p* < 0.027). The average IOP at 24 months postoperatively was 11.74 ± 2.64 mmHg and was significantly improved from preoperative IOP (*p* = 0.000063).

**Fig. 6:**
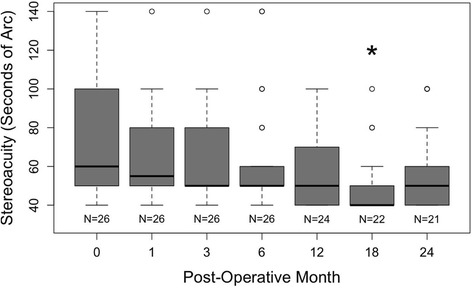
Box-and-whiskers plot of the stereoacuity of patient eyes. The upper and lower extremities of the box represent the 75 and 25th percentiles, the bar within the box represents the median, the whiskers represent the full extent of the data ranges, and the points represent outliers. The star denotes statistical significance compared to preoperative values

Stereopsis testing, as measured by Randot stereoscopic tests, is shown in Fig. 6. Remarkably, stereoacuity improved after the LaserACE procedure. This was statistically significant at 18 months postoperatively (49.1 ± 16.9 s of arc; *p* = 0.012). Preoperatively, mean stereoacuity was 75.8 ± 29.3 s of arc, which improved to 58.6 ± 22.9 s of arc at 24 months, but was not statistically significant.

### Patient satisfaction

**Fig. 7:**
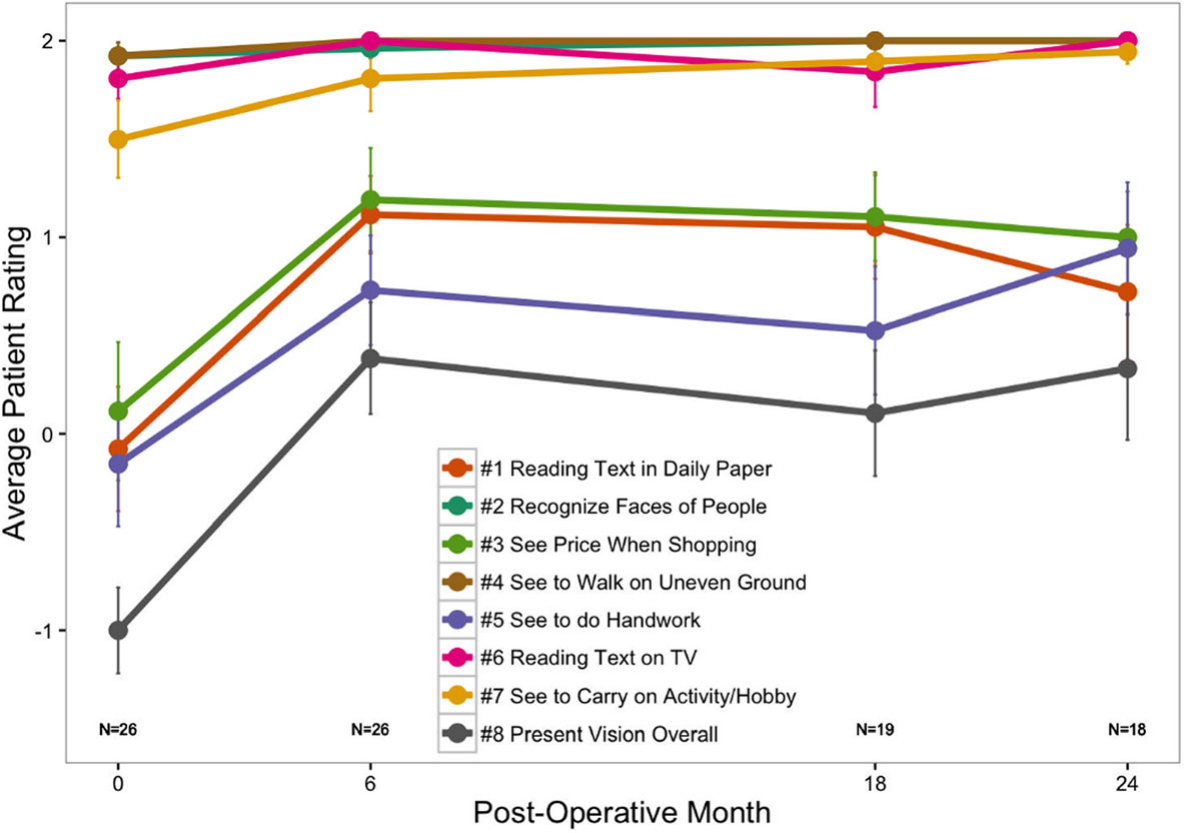
Average participant ratings from the CatQuest 9SF survey. Responses ranged from +2, indicating no difficulty, to −2, indicating great difficulty. Error bars represent mean ± SE

The results of the CatQuest 9SF patient-reported visual function survey are shown in Fig. 7. Satisfaction scores ranged from +2 indicating very satisfied to −2, very dissatisfied. The mean patient satisfaction score and standard error (SE) was −1.00 (SE = 0.22) preoperatively, significantly improving to 0.33 (SE = 0.36) at 24 months postoperatively (*p* = 0.000016). Patients indicated reduced difficulty in areas of near vision, and were overall satisfied with the procedure. Responses ranged from +2 indicating no difficulty to −2, great difficulty. The largest improvement in visual function, as reported by patients, was during their handwork. This improved from a mean rating of −0.15 (SE = 0.32) preoperatively to 0.94 (SE = 0.34) at 24 months postoperatively (*p* = 0.0052). Patients also rated large improvements in their visual function when reading text in daily paper and seeing prices while shopping at 24 months postoperatively. These ratings were all statistically significant at all time points postoperatively (*p* < 0.025). Patients rated very little difficulty in areas of distance vision preoperatively, however they all also reported improvement in distance vision as well by 24 months postoperatively.

**Fig. 8:**
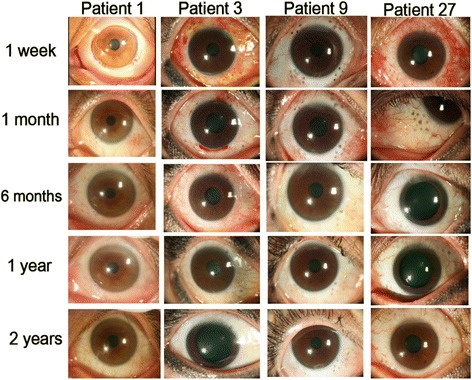
Serial photographs of representative patients from postoperative 1 week to 2 years

Representative photographs of postoperative patient eyes are shown in Fig. 8. During the postoperative period, the most common complaint was mild pain, which relieved within 24 h. Some patients experienced mild tearing, which decreased significantly after 1 week. Very little to no red eye was reported, and was limited to 1 day postoperatively. No ocular emergencies were reported. Two patients had microperforation with reduction in IOP to 5 mmHg and 8 mmHg, respectively, on post-operative day 1. Both patients were managed with collagen matrix application and a bandage soft contact lens, after which IOP normalized by postoperative day 3 with no further complications. One patient had a peripheral corneal abrasion due to accidental laser ablation to an area not completely covered by the corneal shield. This resolved within 5 days. Throughout the whole course of follow-up, there were no complications such as persistent hypotony, cystoid macular edema, or loss of distant best-corrected visual acuity (BCVA) in any of the participants.

## Discussion

LaserACE aims to restore near and intermediate visual acuity in presbyopes by targeting the rigidity of the sclera overlying the ciliary body in three critical zones of anatomical and physiological significance [7, 8, 12, 16–19]. Limiting treatment to the sclera has several advantages over other more invasive options to treat presbyopia. The cornea remains untouched, as does the visual axis and native crystalline lens. This reduces the risk of vision loss, and allows LaserACE to be performed after or in combination with other procedures. No implants are used, and the surgery does not enter the eye. The procedure neither precludes nor complicates future corneal or cataract procedures. Moreover, for additive affect, LaserACE could potentially be combined with other procedures such as PresbyLasik or accommodative intraocular lenses (IOLs). In addition, unlike scleral expansion bands, there are no implants that may erode or extrude.

The positive results obtained at both near and intermediate, given that this is a minimally invasive procedure, are compelling. Changes in UNVA and DCNVA were statistically significant at each follow-up visit. These results surpass early studies using scleral expansion bands, whose results were inconsistent and unpredictable, with a low level of patient satisfaction [24]. A recent study found that 93% of patient eyes had DCNVA of 20/40 or better with the Visibility scleral implant [25]. Lens hardening in older patients may ultimately limit surgical effect.

Changes in monocular UDVA and CDVA were statistically significant at 3 months for CDVA, and at 3 and 6 months for UDVA. Patients who still met the inclusion criteria but were between 0 and +0.5 spherical equivalent were labelled as ‘emmetropic hyperopes’. This was done to distinguish these patients’ outcomes from the emmetropic myopes since they behave differently when accommodation is restored. We expect that patients with any amount of hyperopia would receive a small benefit in their distance vision, as the improved accommodative ability in these patients after LaserACE could be utilized to correct a small degree of hyperopia [26]. When the ‘emmetropic hyperope’ patients are removed from our analyses, the statistically and clinically significant changes in UDVA and CDVA are eliminated.

Other treatments addressing accommodation in presbyopes include accommodating lenses and femtosecond lens treatments. Accommodating lenses attempt to change the IOL position to facilitate near focus. Results have reportedly been moderate, with mean low-contrast UNVA of 20/47 using the Crystalens [27]. Near vision was better with accommodating IOLs than monofocal IOLs, however it was found in another study that this was at least partly due to depth of focus rather than lens movement [28]. The femtosecond lens treatment used to facilitate the change in the lens shape prior to cataract removal yielded mild improvements at 1 month. Binocular DCNVA of patients at 1 month increased 31 letters from baseline. In addition, these treatments are more invasive than the LaserACE technique, with an increased risk of vision loss of between 1 and 2 lines [29].

All ranges of vision showed improvements in visual acuity after LaserACE, with near tasks showing the largest improvements through 24 months postoperatively. Both DCNVA and UNVA had a similar trend of a peak at 6 months postoperatively, then a slight drop off between 6 and 12 months postoperatively. It is of interest to note that the patients’ UVA and DCVA then begin to improve at 12 months postoperatively and continued to improve through 24 months postoperatively. This may be an indication of neuroadaptation or a rehabilitative effect.

The improvements in stereopsis for our LaserACE patients at 24 months postoperatively were both surprising and remarkable, especially since all other presbyopia treatments performed to date have diminished stereopsis and binocularity [30–32]. Monovision, which is either laser or contact lens induced, intentionally decreases binocularity and stereopsis [30]. Corneal presbyopic correction attempts to create a bifocal cornea, however the side effects include a loss of binocularity and stereopsis [31]. Accommodating IOLs potentially could have limited effects on binocularity and stereopsis, but as the surgery is quite invasive, they may be more appropriate for cataract patients [32].

## Conclusions

Our Taiwan IRB monitored clinical trial of the LaserACE procedure utilizing the Er:YAG laser show promising results for restoring the range of visual performance for near, intermediate, and even far visual tasks in emmetropic presbyopes without touching the visual axis or compromising distance vision. The visual function and visual acuity improvements at 24 months postoperatively were clinically significant. Patient satisfaction was high postoperatively and sustained over 24 months. Unlike other presbyopia treatments, stereopsis was not only preserved, but also improved over 24 months.

## Abbreviations

AL: Anatomical limbus
BCVA: Best-corrected visual acuity
CDVA: Corrected distance visual acuity
DCIVA: Distance corrected intermediate visual acuity
DCNVA: Distance corrected near visual acuity
DCVA: Distance corrected visual acuity
Er:YAG: Erbium: yttrium–aluminum–garnet
ETDRS: Early treatment diabetic retinopathy study
IOL: Intraocular lens
IOP: Intraocular pressure
IRB: Institutional review board
LaserACE: Laser anterior ciliary excision
logMAR: Logarithm of minimum angle of resolution
SD: Standard deviation
SE: Standard error
UDVA: Uncorrected distance visual acuity
UIVA: Uncorrected intermediate visual acuity
UNVA: Uncorrected near visual acuity
UVA: Uncorrected visual acuity
